# Recurrent Non-stroke Central Neurologic Manifestations in Primary Antiphospholipid Syndrome

**DOI:** 10.2478/rir-2022-0016

**Published:** 2022-07-06

**Authors:** Hanxiao You, Jiuliang Zhao, Mengtao Li, Xiaofeng Zeng

**Affiliations:** 1 Department of Rheumatology, Peking Union Medical College Hospital, Peking Union Medical College and Chinese Academy of Medical Sciences, National Clinical Research Center for Dermatologic and Immunologic Diseases (NCRC-DID), Key Laboratory of Rheumatology and Clinical Immunology, Ministry of Education, Beijing 100730, China

**Figure 1:**
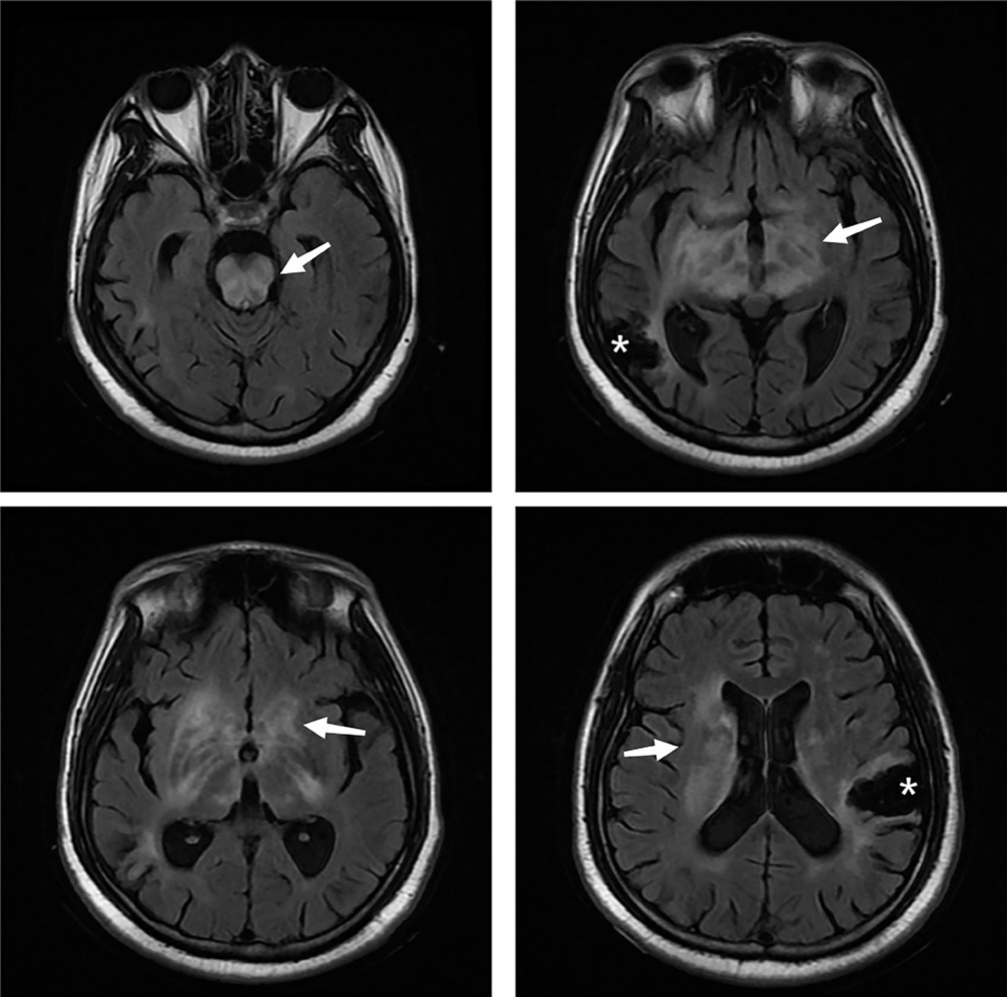
Cranial MRI before treatment: Diffuse abnormal signal in the pons, midbrain, basal ganglia, thalamus and beside the right lateral ventricle (arrows), softened lesions in the right temporal parietal occipital lobe, left frontal lobe and the left cerebellar hemisphere. *obsolete cerebral infarction. MRI, magnetic resonance imaging.

The patient was a 32-year-old male presenting with a 5-year history of gait disturbance and a 2-month history of drowsiness. Initially he had gait disturbance signs and symptoms in July 2013, and cranial computed tomography exhibited multiple low-density shadowson the left cerebellum A diagnosis of cerebral infarction was made and antiplatelet therapy was initiated. Unfortunately, he suddenly developed gait disturbance on the right side and motor aphasia in June 2015. Routine blood examination showed decreased platelet count, 89 × 10^9^/L and positive anticardiolipin antibody (ACL). Tests for antinuclear antibody and anti-extractable nuclear antigen antibodies were negative. Cranial magnetic resonance imaging (MRI) showed new subacute cerebral infarctions in the left frontal and parietal lobes. He was diagnosed with primary antiphospholipid syndrome (APS) cerebral infarction and started on therapy of methylprednisolone (MP) 24 mg daily, hydroxychloroquine, low-molecular-weight heparin, aspirin, and clopidogrel. His symptoms improved and he discontinued all drugs except clopidogrel and corticosteroids (regular tapering) after discharge. However, he became somnolent with unstable walking and urinary incontinence in February 2018, then aphasia and developed swallowing difficulties in April. Laboratory tests revealed triple-positive antiphospholipid antibodies (β2-glycoprotein I (β2GPI), lupus anticoagulant (LA), and ACL). Cranial MRI revealed diffuse abnormal signals in the pons, midbrain, basal ganglia, thalamus, and beside the right lateral ventricle (Figure 1).

APLs associated thrombotic microangiopathy was considered. Then high-dose MP of 1 g/d (intravenously for 3 days) was given on the basis of anticoagulant therapy of low-molecular weight heparin (6000 U/12 h), then followed by 80 mg/d and combined with cyclophosphamide (CTX). Intravenous immunoglobulin 20 g/d for 3 days was also administered.

**Figure 2:**
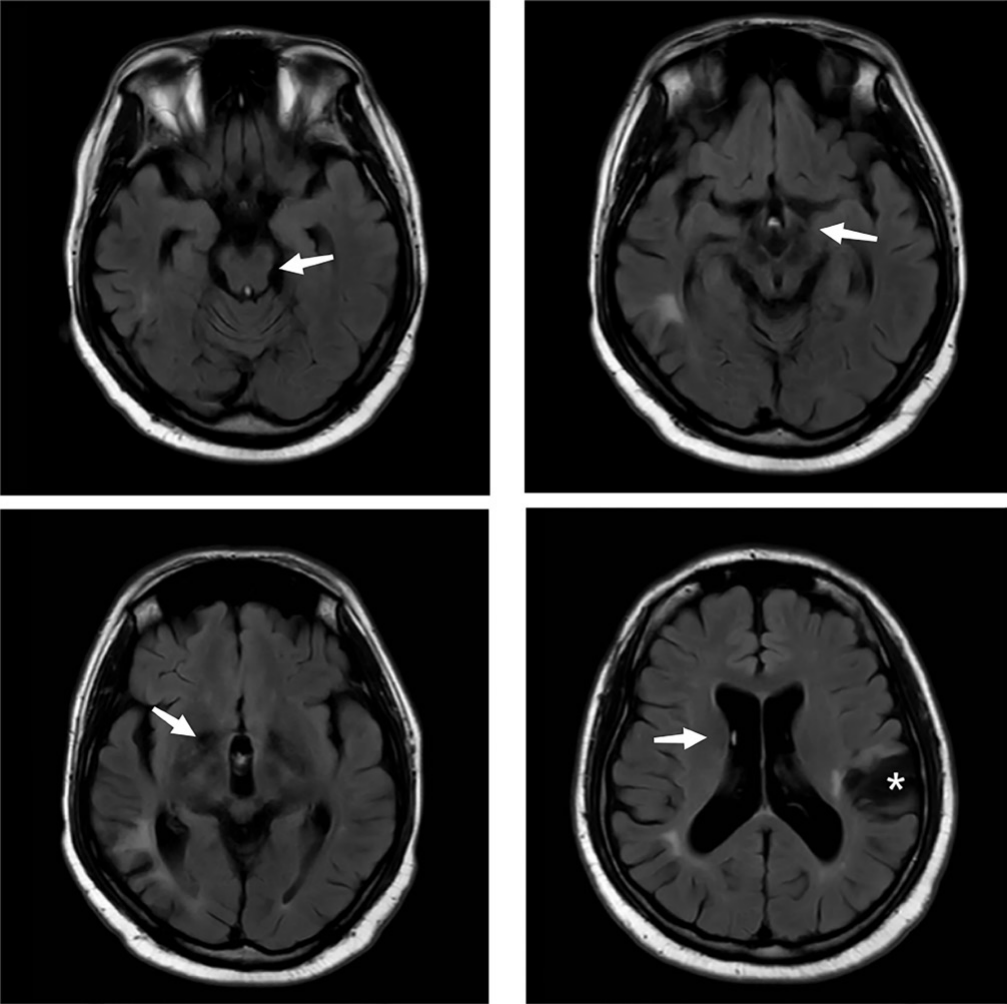
Cranial MRI 6 months after treatment of high-dose glucocorticoids: Most abnormal signals in the bilateral basal ganglia and midbrain disappeared (arrows). *obsolete cerebral infarction. MRI, magnetic resonance imaging.

The patient's consciousness and mental state improved significantly. He was able to perform limb exercises and regained the ability to talk. The patient continued taking prednisone, CTX, hydroxychloroquine, and warfarin after discharge and started rehabilitation exercises. Cranial imaging 6 months later showed that the bilateral basal ganglia and midbrain abnormal signals almost disappeared (Figure 2). There were no new cerebrovascular events during the 3-year follow-up period. In summary, non-stroke central neurologic manifestations in APS patients may be associated with immune-mediated inflammation and thrombotic microangiopathy factors. Anticoagulation combined with intensive immunosuppressive therapy should be considered as treatment.

